# Poisoning by a Ten-Grain Dose of Antipyrin

**Published:** 1888-04

**Authors:** 


### *POISONING BY A TEN-GRAIN DOSE OF ANTIPYRIN.*

S. Peters, M. D., in the *Medical Register* of March 24th, reports the following case :

For a severe headache, of a nervous character, in a lady—Mrs. H.—of about twenty-five years of age, and otherwise healthy, I prescribed two powders (ten grains each) of antipyrin, one to be taken an hour after the first, if needed. She took one about 9.30 P. M., and in two or three minutes she began to experience a "snapping" in her head, along with an itching and burning in the mouth and throat, particularly in the roof of the mouth. This feeling also extended to the eyes, nose and ears, and became so violent that she involuntarily thrust her fingers into her mouth and ears to seek relief. The "snapping" in the head increased in intensity till she became almost frantic, and ran up and down the room screaming, partially losing control of herself, and apprehending acute insanity. Sneezing soon commenced, and became extremely violent, the act being repeated at least fifty times, while the nose and eyes were running a very copious, watery fluid. The turgescence of the mucous membrane was so extreme, that she could not breathe through the nostrils for several hours—indeed, not until the next day. Following all this, there was a stupid, tormenting feeling, with swelling of the nose and eyes, till, exhausted, she finally fell asleep. This sleep was disturbed and tiresome, but the headache proper was relieved. The most violent part of the process continued for only about ten minutes, but recovery was not perfect till the next day.

